# Limb Volume Changes and Activities of Daily Living: A Prospective Study

**DOI:** 10.1089/lrb.2020.0077

**Published:** 2021-06-15

**Authors:** Jae Hyung Park, John Merriman, Abraham Brody, Jason Fletcher, Gary Yu, Eunjung Ko, Alejandra Yancey, Mei R. Fu

**Affiliations:** ^1^ NYU Rory Meyers College of Nursing, New York, New York, USA.; ^2^ Boston College William F. Connell School of Nursing, Chestnut Hill, Massachusetts, USA.

**Keywords:** limb volume changes, daily activities, lymphedema breast cancer

## Abstract

***Background:*** Breast cancer-related lymphedema (BCRL) limits the movements of patients' limbs, which leads to a diminished ability to achieve essential activities of daily living (ADLs). The purpose of this study was to examine the associations between limb volume changes from the baseline before breast cancer surgery and self-reported difficulty in performing ADLs at 12 months following cancer surgery. We hypothesized that a positive association existed between limb volume changes from the baseline and self-reported difficulty in performing ADLs at 12 months following breast cancer surgery.

***Methods and Results:*** The data of the present study were part of a larger study with 140 breast cancer patients recruited before breast cancer surgery and followed up during their first year of treatment. Patients with more than 10% limb volume increase reported more frequent distress in performing 13 ADL items, compared with patients whose limb volume increased by 5%–10%. Regression analysis showed a significant increase in the odds ratio of reporting difficulty in ADLs compared with the group with less than 5% limb volume increase.

***Conclusion:*** Overall, patients with a greater limb volume increase underwent more difficulty performing ADLs. Patients reported more difficulty in performing ADLs even with 5%–10% limb volume increase. Currently, there is no standardized guideline to diagnose BCRL, although previous evidence suggests a limb volume increase greater than 10% as a criterion for BCRL. The findings from the present study suggest a more precise and clinically meaningful criteria for diagnosing BCRL to accommodate those with 5%–10% increase in limb volume.

## Introduction

Breast cancer-related lymphedema (BCRL) is defined as an accumulation of limb fluid within the interstitial space around extremities that leads to the swelling of an affected limb or trunk.^1^ According to a systematic review that included 10 studies and 2192 breast cancer patients, an overall incidence rate of BCRL in the arm was 26%, although the incident rate varies from 0% to 56%, depending on the timing of assessment and the type of procedure that patients underwent.^2–4^ The etiology of BCRL is not fully understood, which cannot explain why some patients develop BCRL, while others do not develop it under the same preventative interventions and similar cancer treatment modalities, and why BCRL develops early in some patients, while others develop it decades after the surgery.^5^ Moreover, no single standardized criterion to diagnose BCRL exists. There are various methods to measure lymphedema, including water displacement, circumferential tape measurement, bioimpedance spectroscopy, and perometric measurement.^6^ Among those methods, perometric measurement using an infrared sensor perometer is the method that shows excellent validity, reliability, and sensitivity in calculating total limb volume.^7,8^ It is known that a limb volume change of 5%–10% by perometric measurement warrants close monitoring or intervention to prevent progression into a limb volume change above 10%, which is a more conservatively accepted criterion among the many criteria for BCRL diagnosis.^9,10^

One potential effect of BCRL is impairment in activities of daily living (ADLs), which include bathing, dressing, toileting, transferring, continence, and feeding.^11–16^ ADLs are sets of essential activities that enable a person to live a minimally independent life; thus, they are important indicators for physical function. With impairments in ADLs, quality of life and independence of living diminish because one cannot function as an independent individual when basic daily living activities cannot be accomplished. Despite the importance of ADLs, studies examining the severity of limb volume change and its impact on ADLs are limited and conflicting.

Research evidences support the negative impact of BCRL and patients' quality of life, including physical function. A study conducted with 50 Canadian women highlighted that there is a negative correlation between limb volume differences above 200 mL and physical function measured in the Short Form 36-Item Health Survey (SF-36).^12^ In a study with 55 Dutch women with breast cancer, arm volume was a predictor for low subscale in physical function and physical role limitation of a 6-item Health Survey (RAND-36).^15^ Similarly, in a study that involved 253 BCRL and non-BCRL patients, BCRL patients showed significantly lower scores on all domains of the European Organization for Research and Treatment of Cancer questionnaire (EORTC-QLQ-C30).^14^ After controlling for covariates, including the number of limb nodes removed and the presence of chemotherapy/radiation therapy, BCRL was significantly related to the lower physical function, role function, cognitive function, and body image.^14^ Also, there was a study that followed 61 women for 5 years after breast cancer surgery, which revealed a positive correlation between BCRL and the degree of difficulty in domestic functioning.^16^ A study with 54 BCRL patients revealed that there is a negative correlation between the disability in the upper extremities and quality of life scores using SF-36.^11^ Nevertheless, there was no correlation between the size of BCRL and SF-36 scores.^11^ It should be noted that SF-36 may not be able to capture the impact of ADLs for breast cancer survivors since it does not include ADLs that are important for breast cancer survivors, such as cooking, making bed, bathing self, dressing self, using knife to cut food, cleaning house, writing or typing, vacuuming, doing laundry, taking care of children, carrying or lifting heavy objects, yard work or gardening, and driving.

Currently, no previous study comprehensively examined individual ADLs. Aforementioned studies instead explored some ADLs as a subset of variables within their questionnaires to assess quality of life, and there was no study that exclusively focused on ADLs. However, quality-of-life measures in the prior research did not reflect comprehensive aspects of ADLs. Furthermore, those studies do not allow the identification of association between the degree of limb volume change and individual ADLs. To the best of our knowledge, no previous studies have examined a relationship between limb volume change and individual ADLs. This study therefore sought to explore the associations between the degree of limb volume increase from baseline before breast cancer surgery and its impact on ADLs at 12 months postsurgery. We hypothesized that patients with more limb volume increase would have greater difficulty in performing ADLs at 12 months postsurgery.

## Materials and Methods

### Design and data source

This study was conducted as a secondary analysis of data from a larger study that is funded by the National Institutes of Health (R21NR012288). The prospective, repeated-measures study explored relationships among limb volume change, BCRL symptoms, and genetic variations of 140 breast cancer patients during their first year of treatment.^17–19^ Patients were assessed before surgery, 4–8 weeks after surgery, and 12 months following surgery. During each visit, investigators measured patients' limb volume and difficulty performing ADLs.

### Ethical consideration

The study was approved by the NYU Langone Institutional Review Board.

### Participants

One hundred forty women with breast cancer were recruited from the Perlmutter Cancer Center in New York City between December 2011 and April 2014. They were enrolled before surgery and followed up for 12 months after surgery. For the duration of the study, 136 patients among 140 patients completed the study, for a 2.9% attrition rate.^19^ More details regarding recruitment and consent process were published.^19^

### Measurement

#### Limb volume

During three visits, investigators measured limb volume by an infrared perometer (Perometry 350S). Currently, the more conservatively accepted criterion for diagnosing BCRL is a 10% or more increase in limb volume, which is calculated by the comparison of limb volume change from the baseline in ipsilateral extremities with the contralateral extremities.^9,10,18^ In the study, limb volume change was calculated using the following formula: Lymphedema = (Ipsilateral Frustum LV_Follow-up_/Ipsilateral Frustum LV_baseline_)/(Contralateral Frustum LV_Follow-up_/Contralateral Frustum LV_baseline_).^18^ Although the widely accepted diagnostic criterion of BCRL is a limb volume change of 10% or more, it is known that even a 5% change in limb volume causes symptoms and discomfort.^20^ In this study, our team explored whether even a 5% increase in limb volume also caused difficulty in performing ADLs. Consequently, the 12-month limb volume change from baseline was categorized into three groups: limb volume changes less than 5%, 5%–10%, and above 10%.

#### Activities of daily living

*Breast Cancer and Lymphedema Symptom Experience Index (BCLE-SEI) Part II* evaluates the symptom distress, that is, the negative impact and suffering evoked by an individual's experience of lymphedema symptoms, including daily living, function, social impact, sleep disturbance, sexuality, emotional/psychological distress, and self-perception.^21,22^ Items for ADL in BCLE-SEI were used to assess self-reported difficulty in ADLs. Patients were given thirteen 4-point Likert scale ADL items specified in Table 1. ADL items can be further classified into self-care and activities that are essential in maintaining independence. Scores from each question item were rated in terms of degrees of difficulty as follows: 0 = no difficulty, 1 = a little, 2 = somewhat, 3 = quite a bit, and 4 = a lot. The total ADL scores were calculated by summing up the total score of each item. The range of the sum of scores from 13 ADL items was 0 to 52; higher scores indicating more difficulty in performing ADLs. Each ADL was also examined in a dichotomized manner by indicating the presence or absence of difficulty. Cronbach's alpha for the 13 ADL items in this sample was 0.81, indicating acceptable reliability.

**Table 1.** Activities of Daily Living

Self-care	Independent daily activity
Bathing self Dressing self	Using a knife to cut food Cooking Writing or typing using a computer Cleaning a house Vacuuming Doing laundry Taking care of children Carrying or lifting heavy objects Yard work or gardening Driving Making a bed

#### Physical symptoms related to lymphedema

BCLE-SEI Part I includes a 5-point Likert-type self-report items to assess occurrence of physical symptoms related to lymphedema, including impaired limb mobility in the shoulder, arm, elbow, wrist, and fingers, arm swelling, breast swelling, chest wall swelling, heaviness, firmness, tightness, stiffness, numbness, tenderness, pain/aching/soreness, stiffness, redness, blistering, burning, stabbing, tingling (pain and needles), hotness, blistering, seroma, limb fatigue, and limb weakness.^21,22^ BCLE-SEI Part I demonstrated a great validity and reliability in identification of difference in symptom occurrence between breast cancer survivors with lymphedema and patients without lymphedema.^19,22^

#### Occupational distress

Patients were asked to answer the question of “How much do your symptoms negatively affect your work outside the home (occupation)?” Responses were dichotomized into the presence and absence of occupational distress.

### Data analysis

Data were analyzed using STATA v16 (StataCorp. 2019. Stata Statistical Software: Release 16. StataCorp LP, College Station, TX). Descriptive statistics and regression analysis were used to report findings. Descriptive statistics were used to calculate frequency, percentage, and *p*-value. For data that are not normally distributed, the median and interquartile range was reported. Regression analysis was used to calculate odd ratios, 95% confidence interval, and *p*-value, referencing the group with less than 5% limb volume change. Dichotomized variables were examined using Fisher's exact test and logistic regression modeling. All tests were conducted at the 0.05 alpha level.

## Results

### Participant characteristics

Table 2 shows baseline demographic and clinical information of the study participants. The mean age of all participants was 52.1 years (standard deviation: 11.11 years). Twenty-one percent of the patients had nine or more physical symptoms of lymphedema. The percentage of patients who underwent lumpectomy and mastectomy with immediate reconstruction was 48.5% and 40.4%, respectively. The median number of limb nodes removed was two. Fifty-five percent of patients reported that they had chemotherapy, and 70.7% of them had adjuvant therapy and 70.1% had radiation therapy. There was no statistically significant sociodemographic difference by limb volume change, among patients with limb volume changes less than 5%, 5%–10%, and above 10%.

**Table 2.** Participant Characteristics

	12-Month LV change from baseline							Total (*n* = 136)	
	LV change <5% (*n* = 105)		LV change 5%–10% (*n* = 22)		LV change >10% (*n* = 9)		*p*-Value		
Age M (SD)	51.7	10.9	54.4	12.7	51.6	10.3	0.581^a^	52.1	11.109
	n	*%*	n	*%*	n	*%*	p*-Value*	n	*%*
Education							0.693^b^		
Associate degree or less	32	30.5%	8	36.4%	5	55.6%		45	33.1%
Bachelor's degree	49	46.7%	10	45.5%	3	33.3%		62	45.6%
Graduate degree	24	22.9%	4	18.2%	1	11.1%		29	21.3%
Marital status							0.124^b^		
Married/partnered	64	61.0%	12	54.6%	4	44.4%		80	58.8%
Divorced/widowed	11	10.5%	6	27.3%	3	33.3%		20	14.7%
Single, never partnered	30	28.6%	4	18.2%	2	22.2%		36	26.5%
Ethnicity							0.665^b^		
Black or African American	21	20.0%	3	13.6%	3	33.3%		27	19.9%
White or Caucasian	64	61.0%	14	63.6%	4	44.4%		82	60.3%
Asian	10	9.5%	3	13.6%	0	0.0%		13	9.6%
Hispanic or Latino	8	7.6%	2	9.1%	2	22.2%		12	8.8%
Other	2	1.9%	0	0.0%	0	0.0%		2	1.5%
Employment							0.343^b^		
Unemployed	16	15.2%	6	27.3%	1	11.1%		23	16.9%
Employed	89	84.8%	16	72.7%	8	88.9%		113	83.1%
Physical symptom counts							0.002^b^		
Healthy (0–3 symptoms)	46	43.8%	5	22.7%	1	11.1%		52	38.2%
At-risk (4–8 symptoms)	44	41.9%	9	40.9%	2	22.2%		55	40.4%
Lymphedema (≥9 symptoms)	15	14.3%	8	36.4%	6	66.7%		29	21.3%
Surgery							0.350^b^		
Mastectomy	11	10.5%	3	13.6%	1	11.1%		15	11.0%
Lumpectomy	53	50.5%	7	31.8%	6	66.7%		66	48.5%
Mastectomy with immediate reconstruction	41	39.1%	12	54.6%	2	22.2%		55	40.4%
Limb node	*n* = 85		*n* = 9		*n* = 6		0.939^c^	*n* = 100	
Number of nodes removed Median, IQR	2	2–4	2	1–5	3	1–7		2	2–4
Chemotherapy	*n* = 54		*n* = 14		*n* = 7		0.494^b^	*n* = 75	
Neoadjuvant therapy	14	25.9%	5	35.7%	3	42.9%		22	29.3%
Adjuvant therapy	40	74.1%	9	64.3%	4	57.1%		53	70.7%
Radiation	*n* = 97		*n* = 21		*n* = 9		0.109^b^	*n* = 127	
No	32	33.0%	6	28.6%	0	0.0%		38	29.9%
Yes	65	67.0%	15	71.4%	9	100.0%		89	70.1%

^a^ Analysis of variance.

^b^ Fisher's exact test.

^c^ *p*-Value of the number of limb nodes removed was calculated by Kruskal–Wallis equality-of-populations rank test, referencing the group with limb volume change less than 5%. *p*-Value is 0.941 and 0.939 for the groups with 5%–10% and above 10% limb volume change, respectively.

IQR, interquartile range; LV, limb volume; M, median; *n*, sample size; SD, standard deviation.

### Sum of ADL scores at 12 months

Table 3 provides the sum of ADL scores at 12 months postsurgery by the percentage of limb volume change. The group with more severe limb volume change at 12 months postsurgery reported more significant distress in performing ADLs. The median sum of the score was 0 (0–2) in the group with limb volume change less than 5%; however, the median sum of the score was 4 (0–10) in the group with limb volume change more than 10%. When adjusting for ties, difference in the median sum of scores in each group was statistically significant (*p* = 0.028).

**Table 3.** Activities of Daily Living Scores

12-Month LV change from baseline	Sum of ADL scores at 12 months postsurgery			
	*n*	Median	IQR	*p*-Value
LV change <5%	105	0	0–2	0.028
LV change 5%–10%	22	1	0–9	
LV change >10%	9	4	0–10	

Sum of ADL scores was calculated by adding an individual score of 13 ADL items in the SEI-BCLE questionnaire.

*p*-Value was calculated by the Kruskal–Wallis equality-of-populations rank test, referencing the group with limb volume change less than 5%.

M, median; *n*, sample size.

### Limb volume and difficulty performing ADLs

Table 4 demonstrates the frequency and percentage of patients who reported having difficulty in performing the 13 ADLs by their limb volume change from baseline to 12 months. For every item, the group with limb volume change over 10% had the highest rate of patients who reported difficulty, followed by groups with 5%–10% and less than 5% limb volume change, respectively.

**Table 4.** Activities of Daily Living and Limb Volume Changes

ADL items	12-Month LV changes from baseline					
	LV change <5% (*n* = 105)		LV change 5%–10% (*n* = 22)		LV change >10% (*n* = 9)	
Cooking (*n* = 101)						
*N*, %	2	2.53%	4	25.00%	2	33.33%
*p*-Value	0.001					
Using a knife to cut food (*n* = 102)						
*N*, %	3	3.75%	4	25.00%	2	33.33%
*p*-Value	0.003					
Writing or typing or using computer (*n* = 102)						
*N*, %	11	13.75%	5	31.25%	3	50.00%
*p*-Value	0.030					
Cleaning house (*n* = 100)						
*N*, %	11	14.10%	7	43.75%	4	66.67%
*p*-Value	0.001					
Vacuuming (*n* = 97)						
*N*, %	7	9.33%	7	43.75%	4	66.67%
*p*-Value	<0.001					
Doing laundry (*n* = 101)						
*N*, %	4	5.06%	6	37.50%	4	66.67%
*p*-Value	<0.001					
Bathing self (*n* = 102)						
*N*, %	5	6.25%	4	25.00%	2	33.33%
*p*-Value	0.016					
Taking care of children (*n* = 67)						
*N*, %	1	1.72%	0	0.00%	2	100.00%
*p*-Value	0.003					
Carrying or lifting heavy objects (*n* = 100)						
*N*, %	30	37.97%	11	68.75%	4	80.00%
*p*-Value	0.020					
Yard work or gardening (*n* = 85)						
*N*, %	8	11.94%	2	14.29%	3	75.00%
*p*-Value	0.011					
Dressing self (*n* = 101)						
*N*, %	7	8.86%	5	31.25%	2	33.33%
*p*-Value	0.018					
Driving (*n* = 99)						
*N*, %	3	3.85%	2	13.33%	3	50.00%
*p*-Value	0.002					
Making bed (*n* = 102)						
*N*, %	13	16.25%	6	37.50%	3	50.00%
*p*-Value	0.030					

*p*-Value calculated by Fisher's exact test.

ADLs, activities of daily living; *N*, count of corresponding participants; *n,* sample size.

Table 5 describes the odds ratio of having difficulty performing each ADL item and an occupational distress item. In particular, patients with a limb volume change greater than 10% versus those with change less than 5% reported 19 times greater odds of difficulty cooking (odds ratio [OR], 19.25; 95% confidence interval [CI 2.127–174.194]), 19 times greater odds of difficulty vacuuming (OR, 19.43; 95% CI [3.003–125.704]), and almost 38 times greater odds of difficulty doing laundry (OR, 37.50; 95% CI [5.214–269.705]). Occupational distress was significant in groups with more than 10% limb volume change (OR, 18.84; 95% CI [1.686–157.313]), but this model did not reach an overall statistical significance. In this logistic regression analysis, taking care of children item was omitted due to a zero frequency in subjects in 5%–10% limb volume change and collinearity in the group with limb volume change above 10%.

**Table 5.** Impaired Activities of Daily Living and Limb Volume Changes

12-Month LV change from baseline	Cooking			
	OR	S.E.	*p*-Value	95% CI
LV change 5%–10%	12.83	11.81	0.006	2.115–77.877
LV change >10%	19.25	21.63	0.008	2.127–174.194
Intercept	0.03	0.02	<0.001	0.006–0.106
	*Cleaning house*			
*12-Month LV change from baseline*	*OR*	*S.E.*	p*-Value*	*95% CI*
LV change 5%–10%	4.74	2.84	0.010	1.462–15.350
LV change >10%	12.18	11.27	0.007	1.987–74.674
Intercept	0.16	0.05	<0.001	0.087–0.311
	*Bathing self*			
*12-Month LV change from baseline*	*OR*	*S.E.*	p*-Value*	*95% CI*
LV change 5%–10%	5.00	3.70	0.029	1.174–21.297
LV change >10%	7.50	7.36	0.040	1.095–51.347
Intercept	0.07	0.03	<0.001	0.027–0.165
	*Yard work or gardening*			
*12-Month LV change from baseline*	*OR*	*S.E.*	p*-Value*	*95% CI*
LV change 5%–10%	1.23	1.05	0.809	0.232–6.524
LV change >10%	22.13	26.87	0.011	2.046–239.201
Intercept	0.14	0.05	<0.001	0.065–0.284
	*Making bed*			
*12-Month LV change from baseline*	*OR*	*S.E.*	p*-Value*	*95% CI*
LV change 5%–10%	3.09	1.85	0.059	0.956–9.999
LV change >10%	5.15	4.49	0.060	0.935–28.410
Intercept	0.19	0.06	<0.001	0.107–0.351
	*Carrying or lifting heavy objects*			
*12-Month LV change from baseline*	*OR*	*S.E.*	p*-Value*	*95% CI*
LV change 5%–10%	3.59	2.11	0.029	1.137–11.356
LV change >10%	6.53	7.46	0.100	0.697–61.242
Intercept	0.61	0.14	0.034	0.389–0.964
	*Using a knife to cut food*			
*12-Month LV change from baseline*	*OR*	*S.E.*	p*-Value*	*95% CI*
LV change 5%–10%	8.56	7.05	0.009	1.700–43.051
LV change >10%	12.83	13.44	0.015	1.648–99.906
Intercept	0.04	0.02	<0.001	0.012–0.123
	*Vacuuming*			
*12-Month LV change from baseline*	*OR*	*S.E.*	p*-Value*	*95% CI*
LV change 5%–10%	7.56	4.85	0.002	2.149–26.566
LV change >10%	19.43	18.51	0.002	3.003–125.704
Intercept	0.10	0.04	<0.001	0.047–0.224

	Driving			
12-Month LV change from baseline	OR	S.E.	p-Value	95% CI
LV change 5%–10%	3.85	3.70	0.161	0.585–25.297
LV change >10%	25.00	25.17	0.001	3.476–179.803
Intercept	0.04	0.02	<0.001	0.013–0.127
	*Dressing self*			
*12-Month LV change from baseline*	*OR*	*S.E.*	p*-Value*	*95% CI*
LV change 5%–10%	4.68	3.13	0.021	1.260–17.351
LV change >10%	5.14	4.90	0.085	0.796–33.247
Intercept	0.10	0.04	<0.001	0.045–0.211
	*Writing or typing or using computer*			
*12-Month LV change from baseline*	*OR*	*S.E.*	p*-Value*	*95% CI*
LV change 5%–10%	2.85	1.79	0.096	0.830–9.792
LV change >10%	6.27	5.51	0.037	1.121–35.106
Intercept	0.16	0.05	<0.001	0.084–0.301
	*Doing laundry*			
*12-Month LV change from baseline*	*OR*	*S.E.*	p*-Value*	*95% CI*
LV change 5%–10%	11.25	8.19	0.001	2.701–46.864
LV change >10%	37.50	37.75	<0.001	5.214–269.705
Intercept	0.05	0.03	<0.001	0.020–0.146
	*Difficulty working outside of home (occupation)*			
*12-Month LV change from baseline*	*OR*	*S.E.*	p*-Value*	*95% CI*
LV change 5%–10%	1.63	1.08	0.462	0.444–5.967
LV change >10%	16.29	18.84	0.016	1.686–157.313
Intercept	0.25	0.07	<0.001	0.137–0.441

Odds ratio was calculated by referencing the LV change <5% group.

Taking care of children items were omitted due to a zero frequency in subjects in the LV change 5%–10% group and collinearity in the LV change >10% group.

CI, confidence interval; OR, odds ratio; S.E., standard error.

## Discussions and Conclusions

This study was conducted to explore the association between the limb volume change and difficulty performing ADLs in patients recovering from breast cancer surgery. We found that patients with more limb volume increase suffered greater odds of having difficulty in performing 13 ADL items. Thus, the result supported the aforementioned hypothesis that limb volume change would be positively associated with difficulty performing ADLs.

Notably, the group with limb volume increase above 10% had the greatest difficulty in performing ADLs. However, patients in the 5%–10% limb volume change group also reported difficulty performing ADLs. Although the most conservatively accepted criterion for BCRL is limb volume increase above 10%,^9,10^ even a 5%–10% difference in limb volume causes symptoms of BCRL and exerts a negative impact on patients' ADL.^19^ Taken together, results of the study suggest the consideration of 5%–10% difference in limb volume as a positive diagnosis of lymphedema; further standardization of BCRL diagnosis criteria is needed.^20^

The present study was worthwhile in that it was the first study to explore exclusively on various ADL items. There have been studies to look at physical function variables within the quality-of-life questionnaires, but it was only a minor part of their outcome variables that do not receive much attention. Nevertheless, this study furthered the numbers and types of ADL items to assess more comprehensive aspects of difficulty in performing ADLs in breast cancer patients. Those studies also dichotomized their patients into the absence and presence of BCRL based on medical diagnosis, while this study classified participants into three categories based on limb volume change, which allowed for a more detailed analysis.

There are limitations to this study. First, this study was conducted in a single cancer center in Manhattan, and the majority of participants were white, which may limit the generalizability. Second, there were only nine patients who had limb volume changes over 10%. However, more than 22.7% of total patients have limb volume change above 5%, and multiple patients among the subset reported impaired ADL. Third, not all six domains of ADLs were explored in this study. Toileting, transfer, continence, and feeding were not included among the ADLs evaluated in the present study. As a whole, the present study warrants further studies to generate findings that are more generalizable with all six domains of ADLs. Because this preliminary finding was promising, a further study with a larger sample size needs to be conducted to confirm the finding.

BCRL is the one aspect of long-term sequela that is not fully understood. Diagnostic criteria are not standardized, and different investigators use different methods to determine the presence of BCRL in breast cancer patients, which causes confusion and delays in delivering interventions to prevent or alleviate BCRL. In this current uncertainty and discordance, more evidence on creating diagnostic criteria needs to be collected. Knowing how much increase in limb volume causes perceived discomfort in ADLs can significantly contribute to establishing robust criteria and guidelines for practice. These days, people live longer than any generation that existed, and the death rate of breast cancer patients is lower than before.^23^ Breast cancer patients are considerably younger and have a longer life span than patients with other types of cancer, which put more emphasis on the quality of life in their life after the treatment phase.^24^ Besides, the mean age for 136 participants in this study was 52.1. In the next 10 years, they will be seniors whose ability to perform ADLs is of utmost importance in living independent lives. In light of the above mentioned, a further study needs to be performed.
